# Enhanced passive safety surveillance of the quadrivalent inactivated split-virion influenza vaccine (IIV4) in Finland during the 2019/20 influenza season

**DOI:** 10.1186/s12889-021-10378-8

**Published:** 2021-02-15

**Authors:** Anne-Laure Chabanon, Sophie Wague, Annick Moureau, Markku Nissila, Laurence Serradell

**Affiliations:** 1 Sanofi Pasteur, Siège Mondial Campus Sanofi Lyon, 14 Espace Henry Vallée, 69007 Lyon, France; 2 Global Biostatistical Sciences, Sanofi Pasteur, 1541 Avenue Marcel Mérieux, 69280 Marcy l’Etoile, France; 3 Terveystalo Biobank and Clinical Research, Humalistonkatu 7b, 20100 Turku, Finland

**Keywords:** Seasonal influenza, Vaccine, IIV4, Influenza season 2019/20, Vaxigrip Tetra, Quadrivalent split-virion inactivated influenza vaccine

## Abstract

**Background and aims:**

The Enhanced Passive Safety Surveillance is a requirement of the European Medicines Agency (EMA) for seasonal influenza vaccines, aiming to rapidly detect any significant change in frequency or severity of expected reactogenicity or allergic events prior to widespread use of a vaccine in any particular year. The aim of this surveillance was to assess the quadrivalent inactivated split-virion influenza vaccine (IIV4) during routine immunization in Finland, as per the national immunization program for 2019/20. The primary objective was to investigate the suspected adverse drug reactions (ADR) occurring within 7 days following vaccination.

**Methods:**

Passive surveillance of individuals vaccinated with IIV4 was conducted within the first 4 to 6 weeks of the influenza season in Finland. Potential ADRs were reported via phone or posted adverse event forms. The vaccinee reporting rate and ADR reporting rate were calculated and compared with the known or expected safety data in order to identify any change which was clinically significant.

**Results:**

Data were collected from 939 individuals, with 56 reports received for 163 suspected ADRs. Of these, 38 individuals reported 117 suspected ADRs within 7 days following vaccination, corresponding to an ADR reporting rate of 12.46% (95% CI: 10.41, 14.74%); vaccination-site pain, vaccination-site reaction, and pyrexia were the most frequently reported ADRs. The 18-to-65 years of age category had an ADR reporting rate of 12.56%, the over-65 years of age category had an ADR reporting rate of 16.22%, and no ADRs were reported for individuals aged 6 months to 18 years. No serious suspected ADRs were reported at any time post-vaccination, and the ADR rates were comparable to those reported for IIV4 in the 2018/19 seasonal assessment. The frequency of suspected ADRs was generally aligned with those reported in the Summary of Product Characteristics (SmPC), with the exception of asthenia, somnolence, and erythema, which were slightly higher. No reporting pattern by type, frequency, or severity was identified for the suspected ADRs.

**Conclusions:**

No clinically significant changes in what is known or expected for IIV4 was reported for the 2019/20 season, which supports the overall safety profile.

## Introduction

Influenza viruses are constantly evolving, and due to this antigenic drift, immunity as a result of prior infections or vaccinations does not provide lasting protection against the virus, which results in seasonal epidemics [1] and necessitates annual update of the vaccine strains and annual vaccination [2]. Therefore, conducting annual safety surveillance on seasonal influenza vaccines is important, and a requirement for the EMA. The safety surveillance enables rapid detection of adverse events (AE), identifying any significant change in frequency or severity of expected reactogenicity or allergic events that could be intrinsic to the vaccine, prior to widespread use of the vaccine in any particular year [3]. Enhanced Passive Safety Surveillance (EPSS) is one of the surveillance methods used for monitoring ADRs [3]. The use of a passive surveillance system in combination with physician reporting of ADRs has been shown to improve the rates of AE reporting [4, 5].

In 2016, a quadrivalent inactivated split-virion influenza vaccine (IIV4; Vaxigrip Tetra®, Sanofi Pasteur) was licensed in the European Union (EU) for use in adults and children from 6 months of age, offering broad protection against influenza through the inclusion of two influenza A and two influenza B virus strains [6]. An EPSS conducted in the northern hemisphere (NH) 2017/18 influenza season assessed the ADR rates for a trivalent split-virion inactivated influenza vaccine (IIV3; Vaxigrip Tetra®, Sanofi Pasteur), an intradermally administered IIV3 (IIV3-ID; Intanza® 15 μg, Sanofi Pasteur) and IIV4, demonstrating consistent safety findings [7]. Therefore, this current surveillance aims to address the requirements of the EPSS for IIV4 during routine immunization, as per the national immunization program in Finland for the influenza season 2019/20.

## Methods

### Study design, population, and setting

Between October 4, 2019 and November 26, 2019 an EPSS was conducted to examine ADRs associated with the IIV4 vaccination within eight participating sites in Finland. The EPSS current interim guidance for seasonal influenza vaccines in the EU recommends a system able to detect ADR normally expected to be common (ie, with a frequency ≥ 1%) and allows the vaccinee or their carer to report on any AEs [3, 6]. Consequently, this surveillance aimed to include 1000 people 6 months of age or older who had received IIV4 from their healthcare professional (HCP) within 4 to 6 weeks following the start of the influenza vaccination season. This population size provided a > 99% probability of collecting ≥1 report of a common AE, which could be a proxy for more severe reactions.

### Vaccine formulation

The IIV4 vaccine contained 15 μg hemagglutinin per strain of: A/Brisbane/02/2018 (H1N1)pdm09-like strain (A/Brisbane/02/2018, IVR-190); A/Kansas/14/2017 (H3N2)-like strain (A/Kansas/14/2017, NYMC X-327); B/Colorado/06/2017-like strain (B/Maryland/15/2016, NYMC BX-69A); B/Phuket/3073/2013-like strain (B/Phuket/3073/2013, wild type) within a 0.5 mL dose. All strains were propagated in fertilized chicken eggs from healthy chicken flocks [6].

### Endpoints

The primary endpoint was the suspected ADRs occurring within 7 days following routine vaccination with IIV4, during the NH 2019/20 influenza season. Secondary endpoints included: suspected ADRs occurring within 7 days following routine vaccination with IIV4, according to the pre-defined age groups, and serious suspected ADRs after vaccination with IIV4 at any time following vaccination. Each of these endpoints were summarized as estimated reporting rates. The reporting rates of suspected ADRs observed during NH influenza season 2019/20 was compared with reporting rates of suspected ADRs observed in NH influenza season 2018/19 for IIV4 vaccines (which was conducted with the same EPSS design), and with frequencies documented in the SmPC.

An exploratory endpoint was included, assessing any potential safety signal(s) detected during the weekly EPSS data review. Safety signals included any reaction which could be causally linked to vaccine exposure and has not previously been known or documented, which could affect the health of the vaccinee [8]. These were evaluated during a weekly cumulative review of all EPSS reported cases, as per the internal Sanofi Pasteur routing pharmacovigilance process.

### Study conduct and data collection

The HCPs were selected from private practices (one main site and seven satellite sites) before the influenza season based on their potential use of the IIV4 vaccine, their influenza vaccination capacities, and estimated representation of all age groups in the routine vaccination population. Vaccination information was recorded by the HCP in real time (or on the same day), in a web portal using the electronic data capture system. Since vaccination followed routine practice, and the goal of an EPSS is to generate data as early as possible, the first 1000 people were enrolled, and no enrollment quota were used.

Any individual receiving IIV4 at the participating sites was given instructions by the HCP to report any suspected ADR, especially those occurring within 7 days of having received the vaccine, either by telephone or postal service. To aid this reporting, vaccinees (or their parent/guardian) received a vaccination card (VC) with the unique vaccine identification number and contact details for ADR reporting by telephone (Sanofi Pasteur Pharmacovigilance [PV] Department), as well as an adverse event form containing general information to fill out regarding a possible ADR (as per routine PV process). The form contained pre-specified outcomes, as well as a free text section in which the ADRs were reported to ensure all relevant data were captured. If completed, this was to be sent to Sanofi Pasteur by post with the pre-paid envelope provided. Due to the nature of the reporting form being free-text, the ADR have been reported verbatim, and no formal definitions have been applied to define particular events.

Data collection and processing for ADRs was conducted by the Sanofi Pasteur PV Department. Verbatim ADRs were entered in the PV database, coded with Medical Dictionary for Regulatory Activities (MedDRA) terminology (version 22.1), and processed according to routine PV processes. Vaccine coverage, as well as suspected ADR reporting data, was evaluated on a weekly basis for signal detection purposes by Sanofi Pasteur. Any ADR recognized as being of interest was analyzed separately as the Pharmacovigilance Risk Assessment Committee (PRAC) ADR. The PRAC ADR of interest are those ADR which are usually solicited within influenza vaccination clinical trials [3], and so rates may be compared with the ADR rates listed within the SmPC. Reports received outside the EPSS period were handled as routine spontaneous reports but were not included in the analysis.

### Statistical methods

Descriptive statistics were used to summarize the data, including the vaccine reporting rate and ADR reporting rate, with associated two-sided 95% confidence intervals (CI). The vaccine reporting rate (number of vaccinees who reported at least one suspected ADR divided by the total number of VCs distributed) and ADR reporting rate (number of suspected ADRs divided by the total number of VCs distributed) were generated in order to identify a clinically significant change, compared with the known or expected safety data.

Data were summarized cumulatively by age group, separated by ADRs occurring ≤7 or > 7 days after vaccination, as well as by seriousness and severity. Potential reactogenicity was assessed by comparing the previous reporting rates obtained in the EPSS NH influenza season 2018/19 and the reporting rates recorded in the SmPC (as per clinical trial findings), with the reporting rates observed in the current EPSS NH influenza season 2019/20. Any observed reporting rates that were higher than the upper limit of the 95% CI of the previous year’s estimate were considered significantly higher.

## Results

Data were collected from 939 individuals vaccinated with IIV4 over the course of 2 months. In total, 56 reports were received for 163 suspected ADRs. The overall vaccinee reporting rate was 5.96% (95% CI: 4.45, 7.48%) and the overall ADR reporting rate was 17.36% (95% CI: 14.99, 19.94%). The time to ADR onset was known for 117 (71.78%) ADRs, and the duration of ADR was known for 79 (48.47%) of the 163 ADRs reported.

For the primary endpoint, 38 people who received the IIV4 vaccine reported 117 suspected ADRs within 7 days following vaccination (Table 1). This corresponds to an ADR reporting rate of 12.46% (95% CI: 10.41, 14.74%). Of the 117 suspected ADRs, 87.18% (102/117) occurred on the same day or the day following vaccination, and none of the ADRs with known duration were reported as having occurred more than 7 days after vaccination. Of the 79 ADRs with known duration, 81.01% (64/79) were resolved within 3 or fewer days, and 18.99% (15/79) resolved within 4 to 7 days. The most frequently reported ADRs from Day 1 were vaccination-site pain, vaccination-site reaction, and pyrexia; and at Day 7, influenza, pyrexia, and vaccination-site erythema.

**Table 1:** Adverse drug reaction rates stratified by age, reported within 7 days of vaccination

	VC distribution (n) (%)	Vaccinees reporting rate (n)	Suspected reactions (n)	% Suspected reactions	95% CI
(All ages)^a^					
ADR		38	117	12.46	10.41, 14.74
PRAC^b^		36	77	8.20	6.53, 10.14
Other^c^		21	40	4.26	3.06, 5.76
6 months to 6 years of age ADR	1 (0.1)	0	0	0.0	0.00, 97.50
6–13 years of age ADR	7 (0.7)	0	0	0.0	0.00, 40.96
13–18 years of age ADR	9 (1.0)	0	0	0.0	0.00, 33.63
18–65 years of age ADR	884 (94.2)	37	111	12.56	10.44, 14.92
>65 years of age ADR	37 (3.9)	1	6	16.22	6.19, 32.01

^a^Age information was missing for one participant

^b^PRAC ADRs of interest for this EPSS are defined according to the ‘Interim guidance on enhanced safety surveillance for seasonal influenza vaccine in the EU’ adopted by PRAC on April 10, 2014 (EMA/PRAC/222346/2014)

^c^Other ADRs are events that do not fall into PRAC ADRs of interest or identified/potential risk

Abbreviations: *ADR* adverse drug reaction; *CI* confidence interval; *EMA* European Medicines Agency; *EPSS* Enhanced Passive Safety Surveillance; *PRAC* Pharmacovigilance Risk Assessment Committee; *VC* vaccination card

The PRAC ADRs of interest accounted for 112 of the 163 ADRs reported (68.71%) and were included in 56 reports. The most frequently reported PRAC ADRs of interest were vaccination-site pain with 27 events (2.88% [95% CI: 1.81, 3.94%]) included in 27 reports, myalgia with nine events (0.96% [95% CI: 0.34, 1.58%]) included in nine reports, and pyrexia and vaccination-site erythema, which both had six events (0.64% [95% CI: 0.13, 1.15%]) included in six reports.

Assessing the secondary endpoints, separating the ADR rates by age group, the ADR rate within 7 days of vaccination for the 18-to-65 years of age category was 12.56% (95% CI: 10.44, 14.92%), and 16.22% (95% CI: 6.19, 32.01%) for the over-65 years of age group. No ADRs were reported for children aged 6 months to 18 years of age. No serious suspected ADRs were reported at any point post-vaccination. All reported suspected ADRs for the NH influenza season 2019/20 were classified as either mild (79, 8.41% [95% CI: 6.72, 10.38%]), moderate (42, 4.47% [95% CI: 3.24, 6.00%]), or unknown (42, 4.47% [95% CI: 3.24, 6.00%]). None of the suspected ADRs reported were specifically classified as severe.

Table 2 shows the comparison of the NH 2018/19 influenza season with the NH 2019/20 influenza season; the overall vaccinee reporting rate for this surveillance (5.96% [95% CI: 4.45, 7.48%]) was found to be similar to the previous season’s rate (5.3% [95% CI: 3.93, 6.71%]. The ADR reporting rate was also similar between this surveillance (17.36% [95% CI: 14.99, 19.94%]) and the previous season’s (16.3% [95% CI: 14.03, 18.71%]). Of note, vaccinee reporting rates for injection-site reactions, a PRAC ADR of interest, were higher in this surveillance (5.54% [95% CI: 4.07, 7.00%]) than for the 2018/19 season (3.6% [95% CI: 2.45, 4.77%]).

**Table 2:** Comparison of reporting rates for all suspected adverse drug reactions between northern hemisphere influenza season 2018/19 and northern hemisphere influenza season 2019/20

	NH influenza season 2018/19 (time to ADR onset)		NH influenza season 2019/20 (time to ADR onset)^**b**^	
Analysis terms (or system organ class) preferred	≤7 days	Total	≤7 days	Total
Total number of VCs distributed		996		939
Total number of vaccinees who reported at least one suspected ADR	28	53	38	56
Vaccinee reporting rate, % (95% CI)	2.8 (1.78, 3.84)	5.3 (3.93, 6.71)	**4.05 (2.79, 5.31)**	5.96 (4.45, 7.48)
Total number of suspected ADRs	76	162	117	163
ADR reporting rate, % (95% CI)	7.6 (6.06, 9.46)	16.3 (14.03, 18.71)	**12.46 (10.41, 14.74)**	17.36 (14.99, 19.94)
Total number of vaccinees who reported at least one PRAC ADR of interest^a^	27	48	36	56
Vaccinee reporting rate, % (95% CI)	2.7 (1.70, 3.72)	4.8 (3.49, 6.15)	**3.83 (2.61, 5.06)**	5.96 (4.45, 7.48)
Total number of PRAC ADRs of interest	47	96	77	112
PRAC ADRs of interest reporting rate, % (95% CI)	4.7 (3.49, 6.23)	9.6 (7.88, 11.64)	**8.20 (6.53, 10.14)**	**11.93 (9.92, 14.17)**

^a^PRAC ADRs of interest for this EPSS are defined according to the ‘Interim guidance on enhanced safety surveillance for seasonal influenza vaccine in the EU’ adopted by PRAC on April 10, 2014 (EMA/PRAC/222346/2014)

^b^Values in bold are those with a higher percentage in the current season compared with the upper limit of the 95% CI of 2018/19

Abbreviations: *ADR* adverse drug reaction; *CI* confidence interval; *EMA* European Medicines Agency; *EPSS* Enhanced Passive Safety Surveillance; *NH* northern hemisphere; *PRAC* Pharmacovigilance Risk Assessment Committee; *VC* vaccination card

Comparing the frequency of suspected ADRs reported in the NH influenza 2019/20 season with the ADR recorded in the SmPC (Table 3), the rates of asthenia, somnolence, and erythema were slightly above the frequency provided in the SmPC (> 0.1% for all three ADRs vs > 0.01 to < 0.1% for the SmPC, respectively). All other listed ADRs were below or within the expected reporting frequencies. The safety analysis of suspected ADRs did not identify any reporting pattern by type, frequency, or severity.

**Table 3:** Comparison of reporting rates for all suspected adverse drug reactions in the 2019/2020 enhanced passive safety surveillance with the reporting rates recorded in the Summary of Product Characteristics

	Current EPSS NH 2019/2020 EPSS (irrespective of time to ADR onset)^**a**^		Vaxigrip Tetra® (Sanofi Pasteur) SmPC		Higher when compared with SmPC
	MedDRA preferred term	Reported frequency (95% CI)	Corresponding ADR terms	Frequency categories if available in SmPC (eg, common, rare)	
PRAC ADR of interest: decreased appetite	Decreased appetite	0.11 (0.00, 0.59) (in adults)	Appetite lost	Not available in the SmPC for adults and elderly	Not applicable
PRAC ADR of interest: injection-site reactions	Vaccination-site warmth	0.11 (0.00, 0.59) (in adults)	Injection-site warmth	Uncommon (≥0.1 to < 1%) in children 3 to 8 years of age, adults and elderly	No
PRAC ADR of interest: myalgia/arthralgia	Joint lock^b^	0.11 (0.00, 0.59) (in adults)		Not available in the SmPC^c^	Not applicable
	Musculoskeletal stiffness^b^	0.11 (0.00, 0.59) (in adults)		Not available in the SmPC^c^	Not applicable
PRAC ADR of interest: rash	Rash	0.21 (0.03, 0.77)	Rash	Not available in the SmPC for children ≥3 years of age, adults and elderly. Rare (≥0.01 to < 0.1%) for children from 6 to 35 months	Not applicable
Other ADRs: general disorders and administration site conditions	Adverse drug reaction	1.28 (0.56, 2.00) (≥13 years of age to elderly)		Not available in the SmPC^c^	Not applicable
	**Asthenia**	**0.11** **(0.00, 0.59)** **(in adults)**	Asthenia	Rare (≥0.01 to < 0.1%)	Yes
	Inflammation	0.11 (0.00, 0.59) (in adults)		Not available in the SmPC for adults and elderly	Not applicable
	Secretion discharge	0.11 (0.00, 0.59) (in adults)		Not available in the SmPC for adults and elderly	Not applicable
	Sluggishness	0.11 (0.00, 0.59) (in adults)		Not available in the SmPC for adults and elderly	Not applicable
	Swelling^d^	0.21 (0.03, 0.77) (in adults)		Not available in the SmPC^c^	Not applicable
	Tenderness	0.11 (0.00, 0.59) (in elderly)	Injection-site pain	Very common (≥10%) in adults and elderly	No
Other ADRs: infections and infestations	Influenza	0.43 (0.12, 1.09) (in adults)	As with any vaccine, vaccination with Vaxigrip Tetra® (Sanofi Pasteur) may not protect all vaccinees	Not available in the SmPC^c^	Not applicable
Other ADRs: musculoskeletal and connective tissue disorders	Neck pain	0.11 (0.00, 0.59) (in adults)		Not available in the SmPC^c^	Not applicable
Other ADRs: nervous system disorders	**Somnolence**	**0.11** **(0.00, 0.59)** **(in adults)**	Somnolence	Rare (≥0.01 to < 0.1%)	Yes
Other ADRs: psychiatric disorders	Delusion	0.11 (0.00, 0.59) (in adults)		Not available in the SmPC^c^	Not applicable
Other ADRs: respiratory, thoracic, and mediastinal disorders	Wheezing	0.11 (0.00, 0.59) (in adults)		Not available in the SmPC^c^	Not applicable
Other ADRs: skin and subcutaneous tissue disorders	**Erythema**	**0.11** **(0.00, 0.59)** **(in adults)**	Hypersensitivity, allergic reactions such as erythema, urticaria, pruritus, pruritus generalized, dermatitis allergic, angioedema	Rare (≥0.01 to < 0.1%) in adults	Yes
Other ADRs: vascular disorders	Flushing	0.11 (0.00, 0.59) (in adults)	Hot flush	Uncommon (≥0.1 to < 1%)	No

^a^Values in bold are reactions that have higher values in the current study compared with the rates reported in the SmPC

^b^Events considered as equivalent to the PRAC adverse events of special interest ‘myalgia/arthralgia’ (in adults and elderly, the SmPC reporting frequency is very common for myalgia and rare for arthralgia)

^c^Unlisted ADR for all age groups

^d^One ADR was likely to be injection-site swelling, which is a listed event with no information available for the second ADR

Abbreviations: *ADR* adverse drug reaction; *CI* confidence interval; *EPSS* Enhanced Passive Safety Surveillance; *MedDRA* Medical Dictionary for Regulatory Activities; *NH* northern hemisphere; *PRAC* Pharmacovigilance Risk Assessment Committee; *SmPC* Summary of Product Characteristics

No safety signals were detected during the EPSS, therefore the safety signals per batch were not assessed for the exploratory endpoint. In addition, the same batch of vaccine was provided to 938 of the 939 individuals.

## Discussion

Since 2014, the EMA has required annual enhanced safety surveillance monitoring for seasonal influenza vaccines, replacing the small-scale safety and immunogenicity clinical trials previously required from the manufacturers of seasonal influenza vaccines [3, 9]. This EPSS assessed the safety of the IIV4 vaccine at the start of the influenza season 2019/20 in Finland. For the primary EPSS analysis, the ADRs reported within 7 days of vaccination were as expected, with no novel AE detected. The most common ADR reported was vaccination-site pain. The majority of the ADRs were reported within 3 days of vaccination and most were resolved within 3 days. As expected, no serious or severe findings were reported at any stage post-vaccination, the ADRs reported were comparable to those reported in the previous 2018/19 season, and they were in line with what had been reported in the SmPC (with the exception of slight increases compared with the SmPC recorded frequency of asthenia, somnolence, and erythema).

EPSS is advantageous in that it provides a near real-time evaluation of the reactogenicity or allergic events of a seasonal influenza vaccine following annual strain changes, which could indicate more potentially serious risks as the vaccination uptake increases [3]. However, there are challenges associated with this reporting method. Recruiting sufficient participants within the month-long surveillance to accommodate the EPSS requirements is one example. Variations in the surveillance conduct (different countries and changes in the reporting methods) can make examining ADR rates across different years less comparable as well. In addition, the nature of passive reporting does not allow for any control over the timing of ADR reporting, or whether an ADR is reported or not (making under-reporting a possibility).

The 2017/18 IIV4 EPSS reported injection-site reactions, headaches, and fever as the most common adverse reactions, and no safety issues were previously observed for IIV4, which is in line with findings from this current surveillance [7]. The rate of AEs was 2.1%, lower than the rate reported in this current surveillance; however, that may be due to the phone reporting methods previously used [7]. Compared with the 2019/20 surveillance, the reporting rates for the 2018/19 season were similar for both the vaccinee reporting rate (6.0% vs 5.3%), and the ADR reporting rate (17.4% vs 16.3%). Using a combination of posted forms and phone reporting within this surveillance was expected to provide better quality reporting than using a method such as email reporting alone, as instructions had been given to the vaccinee to assist them in completing the necessary information for reporting. The rates of spontaneous reporting in this current surveillance (6.0%) were higher than expected, a distinct advantage of this method, and in line with what has been demonstrated in previous passive surveillance studies [4, 5]. This increased rate of reporting, compared with normal spontaneous reporting, is likely due to HCPs increasing awareness in the vaccinees of the importance of suspected ADR reporting.

Limitations of the surveillance include the reduced population size, which was below the intended 1000 individuals, and fewer individuals over 65 years of age than expected, most likely due to competition with an alternate surveillance. Additionally, the majority of sites were private clinics, which may have impacted recruitment for the surveillance. This is likely also the reason for the demographic imbalance in the participant population (with a higher proportion of participants between 18 and 65 years of age). This imbalance was expected as the majority of the sites, being private clinics, were frequented by individuals of working age. As specified within the national vaccination program in Finland, influenza vaccines are freely administered only at public health clinics to those for whom influenza is an essential health risk or those who gain significant benefit from it, including pregnant women, children 7 years of age or younger, and adults 65 years of age and over. The inclusion of all children under 7 years of age, as well as all adults over 65 years of age within the national vaccination program may further explain the lower proportion of these age groups within the surveillance, as they may be more likely to get a free vaccination from a public health clinic instead. While IIV3 is available to all age groups, nasal spray vaccination is also available for children between 24 months and 5 years of age [10]. The use of nasal spray vaccination with live attenuated virus has risen in this age group, compared with IIV3, as demonstrated with a study assessing vaccination rates in 2-year old children in Finland, which showed that 20% of the cohort were vaccinated with the nasal spray vaccine in 2016–2017, followed by 22% in the 2017–2018 period; whereas 8% of children were vaccinated with IIV3 in the 2016–2017 period and 9% received IIV3 in 2017–2018 [11]. The increasing use of the nasal spray vaccine may further explain the lower proportion of children enrolled for vaccination with IIV4 within this surveillance. To this effect, conducting future EPSS within public practice may provide a cohort of vaccinees of all age groups, instead of primarily including the working age demographic. In this surveillance, all but one individual received a vaccine from the same batch, limiting the ability to investigate the safety across multiple batches.

The inconvenience of returning the adverse event form via post does mean some ADRs could have been missed, and postal strikes in Finland also had an impact on data collection, with post delayed up to 2 weeks. A shortage of the vaccine also resulted in a delay to the start of the campaign. Due to these impediments, enrolment was extended by 1 week, and data collection was extended by a further 2 weeks. Digital reporting and structured questionnaires may increase rates of reporting for future studies.

While passive reporting appears to have been advantageous in this surveillance, with a good rate of ADR reporting, there is generally a lack of control with both the level and timing of passive reporting. The self-reporting nature of the surveillance also meant that none of the suspected ADRs were confirmed by a medical professional, and should any of the ADRs have been complicated in nature, obtaining further detail would have proven difficult. As well as under-reporting, differential ADR reporting (where more serious ADRs and ADRs with a shorter time onset after vaccination are more likely to be reported than minor ADRs) was also a possibility in this surveillance.

In conclusion, the 2019/20 EPSS results did not suggest any clinically significant change in what is known or expected for IIV4, which supports the safety profile of this vaccine and continues to maintain public confidence in influenza vaccination. Changes in the adverse event form distribution method improved the quality of the data reported by the patients compared with the last season, allowing for a better assessment of the reported cases, and contributed to increased reporting. Other improvements, including digital strategies, might further improve both the quality of the data and the reporting stimulation in the future.

## Data Availability

The datasets generated and/or analysed during the current study, including the raw data, are not publicly available in order to safeguard the privacy of participants and the confidentiality and protection of their data, as well as protect commercially sensitive information. Qualified researchers may request access to patient level data and related study documents including the clinical study report, study protocol with any amendments, blank case report form, statistical analysis plan, and dataset specifications. Patient level data will be anonymized and study documents will be redacted to protect the privacy of our trial participants. Further details on Sanofi’s data sharing criteria, including required permissions to access the data, eligible studies, and process for requesting access can be found at: https://www.clinicalstudydatarequest.com/.

## Abbreviations

ADR: Adverse drug reaction
AE: Adverse event
CI: Confidence interval
EMA: European Medicines Agency
EPSS: Enhanced Passive Safety Surveillance
EU: European Union
HCP: Healthcare professional
IIV3: Trivalent split-virion inactivated influenza vaccine
IIV3-ID: Intradermally administered trivalent split-virion inactivated influenza vaccine
IIV4: Quadrivalent split-virion inactivated influenza vaccine
MedDRA: Medical Dictionary for Regulatory Activities
NH: Northern hemisphere
PRAC: Pharmacovigilance Risk Assessment Committee
PV: Pharmacovigilance
SmPC: Summary of Product Characteristics
VC: Vaccination card
